# Unusual Radiographic Presentation of *Pneumocystis* Pneumonia in a Patient with AIDS

**DOI:** 10.1155/2017/3183525

**Published:** 2017-12-07

**Authors:** Brian L. Block, Tejas Mehta, Gabriel M. Ortiz, Sean P. Ferris, Thienkhai H. Vu, Laurence Huang, Adithya Cattamanchi

**Affiliations:** ^1^Division of Pulmonary and Critical Care Medicine, University of California, San Francisco, CA, USA; ^2^SBKS Medical Institute and Research Centre, Gujarat, India; ^3^Division of Hospital Medicine, Priscilla Chan and Mark Zuckerberg San Francisco General Hospital and Trauma Center, San Francisco, CA, USA; ^4^Division of Pathology and Laboratory Medicine, University of California, San Francisco, CA, USA; ^5^Department of Radiology and Biomedical Imaging, Priscilla Chan and Mark Zuckerberg San Francisco General Hospital and Trauma Center and University of California, San Francisco, CA, USA; ^6^HIV, Infectious Diseases, and Global Medicine Division, University of California, San Francisco, CA, USA

## Abstract

*Pneumocystis jirovecii* pneumonia (PCP) typically presents as an interstitial and alveolar process with ground glass opacities on chest computed tomography (CT). The absence of ground glass opacities on chest CT is thought to have a high negative predictive value for PCP in individuals with AIDS. Here, we report a case of PCP in a man with AIDS who presented to our hospital with subacute shortness of breath and a nonproductive cough. While his chest CT revealed diffuse nodular rather than ground glass opacities, bronchoscopy with bronchoalveolar lavage and transbronchial biopsies confirmed the diagnosis of PCP and did not identify additional pathogens. PCP was not the expected diagnosis based on chest CT, but it otherwise fit well with the patient's clinical and laboratory presentation. In the era of combination antiretroviral therapy, routine prophylaxis for PCP, and increased use of computed tomography, it may be that PCP will increasingly present with nonclassical chest radiographic patterns. Clinicians should be aware of this presentation when selecting diagnostic and management strategies.

## 1. Introduction

*Pneumocystis jirovecii* pneumonia (PCP) is a potentially life-threatening infection that most often occurs in immunocompromised hosts. PCP was the most frequent complication of HIV/AIDS in the early phases of the epidemic, and while its incidence has declined substantially in the current era of combination antiretroviral therapy, it remains one of the most common HIV-associated opportunistic infections.

Generally, the diagnosis of PCP is suggested by a subacute illness featuring fever, nonproductive cough, malaise, and progressive shortness of breath [1]. Dyspnea and hypoxemia can be profound. Lung auscultation and chest roentgenogram can both be normal, but computed tomography (CT) of the chest typically demonstrates “ground glass opacities” [2, 3]. This is likely due to the accumulation of cellular debris, fibrin, and organisms within the alveolar spaces [2], as well as interstitial inflammation. Other radiographic patterns of PCP include a predilection for upper lobes (particularly in patients who use aerosolized pentamidine for prophylaxis), a preference for central rather than peripheral zones, and a tendency to form cysts [1, 4, 5]. Several serum tests can be used to help determine the likelihood of PCP, but microscopic visualization of induced sputum or bronchoalveolar lavage fluid specimens remains the gold standard for diagnosis [6].

A previous publication from our institution suggested that the presence of ground glass on high-resolution chest CT (HRCT) is 100% sensitive for PCP in patients with AIDS [7]. In other words, the absence of ground glass opacities on HRCT ruled out the diagnosis of PCP in such patients. Based on these data, our standard clinical practice has been to recommend against additional diagnostic testing and empiric PCP therapy for HIV-infected patients when their chest HRCT does not contain ground glass opacities, even in the setting of a clinical syndrome suggestive of PCP [8].

Here, we report a case of PCP with an unusual radiographic appearance consisting of nodular opacities without ground glass on chest CT. While the clinical syndrome was convincing for PCP, the radiographic presentation made PCP seem unlikely and led to an interruption of PCP therapy.

## 2. Case Presentation

A 57-year-old man was admitted to our hospital with a four-week history of nonproductive cough and dyspnea. His past medical history was notable for type II diabetes mellitus, chronic kidney disease with a baseline creatinine of 1.3 mg/dL, and untreated HIV/AIDS, which had been diagnosed 20 years previously. Prior opportunistic infections included oral candidiasis and cryptococcal meningitis. He was allergic to sulfonamides and had been prescribed dapsone for PCP prophylaxis, but he was not taking it reliably. His most recent known CD4 count and HIV viral load, 11 months prior to admission, were 15 cells/uL and 148,582 copies/mL, respectively.

**Figure 1:**
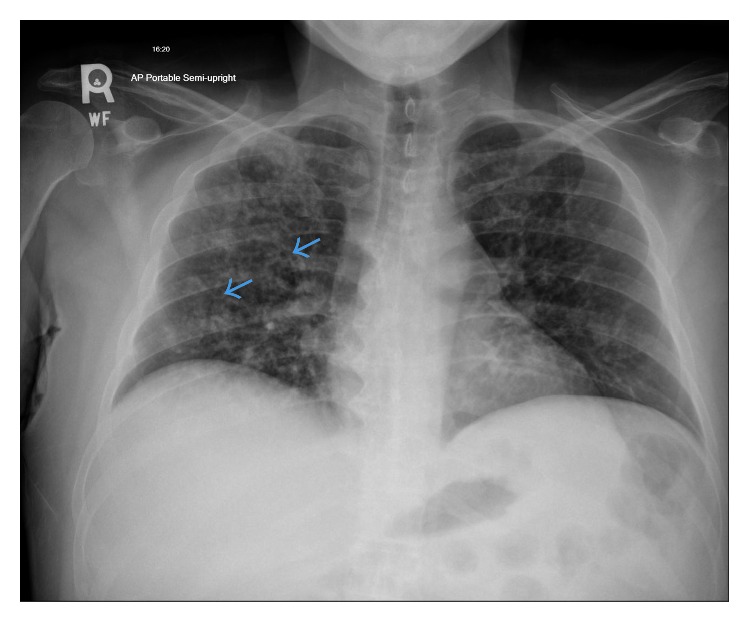
Chest roentgenogram at referring hospital. AP chest radiograph at outside hospital performed 4 days prior. Diffuse micronodules greater in the right lung are seen. Hint of small cavities is noted (blue arrows).

Four days prior to presenting to our emergency department, the patient was admitted to another hospital with the same symptoms of nonproductive cough and shortness of breath. Chest radiograph at that time showed diffuse micronodules, greater in the right lung (Figure 1). There, he was empirically treated for both community-acquired pneumonia and PCP with ceftriaxone, azithromycin, clindamycin, primaquine, and steroids. He was also given fluconazole for thrush. Unfortunately, he left that facility against medical advice after 48 hours. He took no further treatment before coming to our hospital two days later.

**Figure 2:**
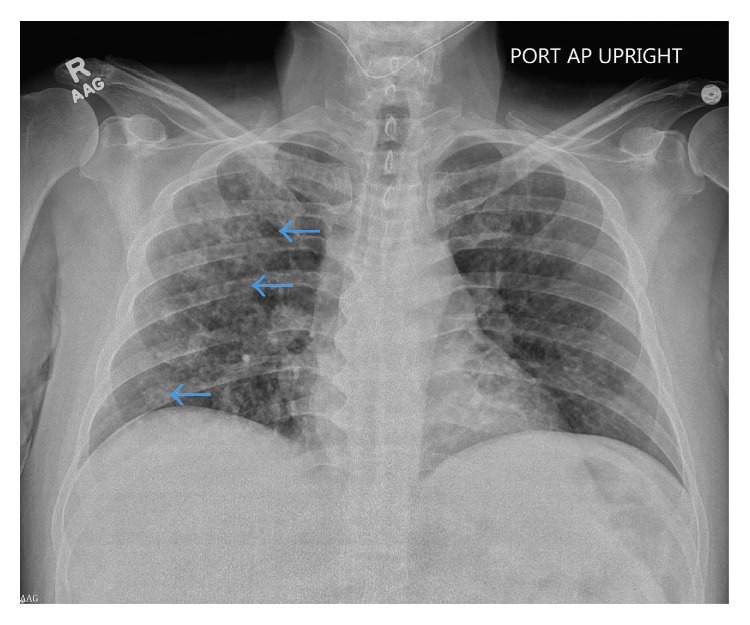
Chest roentgenogram on admission to our hospital. AP chest radiograph at presentation. Again seen are diffuse micronodules with confluent areas in the right upper and lower lungs. Hint of small cavities is again noted (blue arrows).

When he subsequently came to our emergency department complaining of ongoing shortness of breath and cough, temperature was 35.9 degree Celsius, heart rate 119 beats per minute, blood pressure 107/71 mmHg, respiratory rate 18 breaths per minute, and oxygen saturation 96% while breathing ambient air. He was ill-appearing with conjunctival pallor and oral thrush. The remainder of his physical examination was normal, including chest auscultation, which revealed good air movement without wheezing, rales, or rhonchi. Laboratory analysis was notable for white blood cell count of 6,200 cells/uL with 63% neutrophils, 19% lymphocytes, 13% monocytes, and 3% eosinophils, elevated lactate dehydrogenase of 385 u/L, and positive beta-D-glucan of 389 pg/mL (Table 1). Chest roentgenogram revealed diffuse micronodules with confluent areas of nodular opacities (Figure 2). On the basis of these findings, the patient was admitted to our medical service and treated empirically for community-acquired pneumonia with ceftriaxone and doxycycline, for thrush with fluconazole, and for PCP with clindamycin and primaquine.

**Figure 3:**
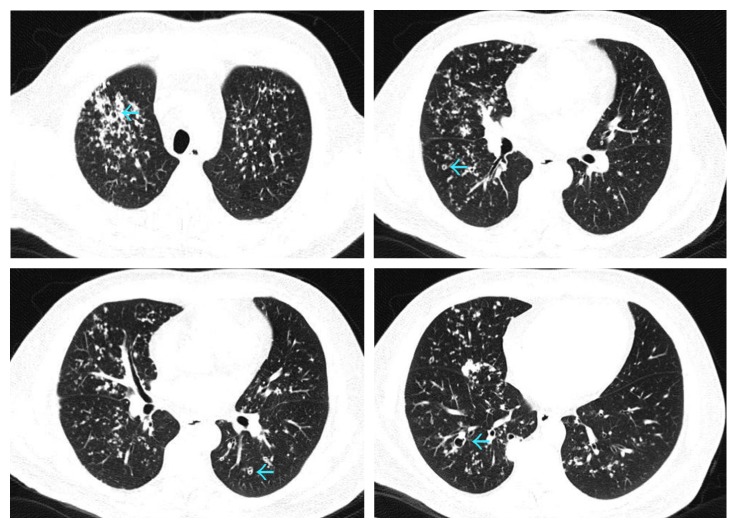
Selected images of computed tomography (CT) scan of the chest on admission to our hospital. Axial images in lung window confirm diffuse micronodules with central cavitation of varying sizes (blue arrows).

**Figure 4:**
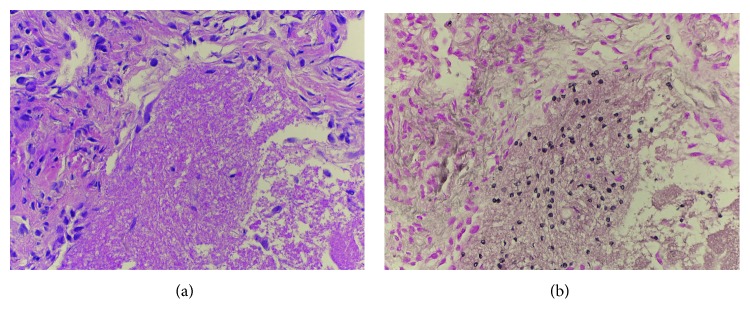
Transbronchial biopsy pathologic results. Pathologic evidence for PCP infection from the patient's transbronchial biopsy specimen. (a) Hematoxylin and eosin- (H&E-) stained section demonstrates classic intra-alveolar eosinophilic foamy exudates. (b) Gomori methenamine silver (GMS) special stain demonstrates nonbudding cysts with thin walls and intracystic capsular dots, consistent with *Pneumocystis jirovecii*-type fungal organisms.

**Figure 5:**
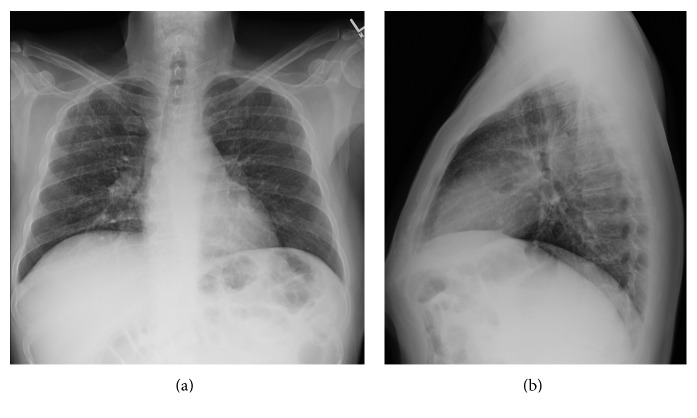
Chest roentgenogram four months after hospitalization. (a) PA and (b) lateral chest radiographs post treatment four months later demonstrated marked improvements.

On hospital day 2, the patient developed worsening shortness of breath with hypoxemia requiring supplemental oxygen. A noncontrast, volumetric chest CT scan (Figure 3) revealed diffuse micronodules with central cavitation and bronchial wall thickening, most suspicious for mycobacterial or fungal infection. Notably, there were no ground glass opacities. Based on this CT and the lack of ground glass opacities, the treating team discontinued PCP therapy, initiated tuberculosis treatment, and consulted the pulmonary service for assistance and consideration of bronchoscopy. The patient underwent bronchoscopy with bronchoalveolar lavage (BAL) and transbronchial biopsy the following day. Pathologic analysis of the transbronchial biopsy specimen demonstrated foamy exudates on hematoxylin and eosin- (H&E-) stained sections, and silver stain revealed *Pneumocystis jirovecii*-type fungal organisms confirming the diagnosis to be PCP [9] (Figure 4). No concurrent infections were found on cultures or pathologic samples (Table 2). The patient was treated with three weeks of clindamycin, primaquine, and prednisone. He was discharged home on hospital day 10 without requiring supplemental oxygen and did well thereafter, with a resolution of radiographic abnormalities on imaging obtained four months later (Figure 5).

## 3. Discussion

Although the incidence of PCP has decreased since the advent of combination antiretroviral therapy in the 1990s, it remains among the most common pulmonary opportunistic infections in patients with AIDS. PCP should be suspected in the clinical setting of subacute dyspnea, nonproductive cough, and fever. While microscopic visualization of bronchoalveolar lavage fluid is the gold standard for diagnosing PCP, chest CT is increasingly being used to make clinical decisions about empiric treatment, and whether to pursue bronchoscopy.

The radiographic appearance of PCP can vary from person to person. The “classic” presentation on plain film is of bilateral, symmetric opacities in an interstitial or alveolar pattern [3, 10]. Chest CT in patients with AIDS who develop PCP most often shows ground glass opacities [2, 4, 11, 12]. However, PCP has also been described to present infrequently with additional findings including cystic lung disease, pneumothoraces, and nodules, among other patterns [2, 3, 13].

It is not entirely surprising that PCP can have a variety of radiographic presentations. As a pathogen that primarily causes disease in the immunosuppressed, PCP often occurs in lungs that have been damaged by prior infections, distorting their architecture. Moreover, it is not uncommon for PCP to co-occur with another infection in an immunocompromised host. In such a circumstance, the radiographic signature of PCP may be subsumed by the coinfecting pathogen.

At our institution where we have extensive experience caring for patients with HIV/AIDS, a chest CT without ground glass is generally thought to “rule out” PCP in patients with AIDS. This is based on a 1997 study of 51 patients with AIDS and a clinical syndrome consistent with PCP but normal chest roentgenogram who underwent HRCT of the chest [7]. In this cohort, the absence of ground glass opacities on chest CT had a perfect negative predictive value for *Pneumocystis* pneumonia (i.e., the presence of ground glass was 100% sensitive for PCP). Knowing these data and subsequent clinical experience, the team treating the described patient was surprised to learn that the patient had PCP, and PCP alone—as the absence of ground glass opacities on chest CT was highly unusual. Indeed, the treating clinicians decided to discontinue PCP-directed therapy on the basis of the CT findings.

While highly atypical, the radiographic appearance of the described patient's PCP is not entirely without precedent. Some of the previously mentioned publications do describe rare instances of PCP presenting as nodules [2–4, 10]. Still, such described presentations were the exception rather than the rule, and over the past 20 years, all cases of PCP that we have seen at our institution have consistently manifested as ground glass opacities on chest CT (when obtained).

It is likely that we, and others, will encounter more cases of PCP with “atypical” radiographic presentations. The increased access to CT scanners means more patients are undergoing chest CT for indications that previously may not have led to such imaging. Our patient, for example, with subacute cough, dyspnea, fevers, and malaise had a clinical history consistent with PCP, and his chest roentgenogram with regions of confluent nodular opacities could have been interpreted as typical of PCP. Based on the inclusion criteria for the aforementioned 1997 study [7] from our institution, our patient would have proceeded directly to sputum induction or bronchoscopy on the basis of his clinical history and chest roentgenogram, without first having undergone chest CT. If that course had been taken, he would have been discovered to have PCP in the setting of a compatible clinical history and imaging (plain film) and his presentation would have been considered entirely typical. We would not have appreciated the exclusively nodular aspect of his disease.

Beyond the increased availability of cross-sectional imaging with chest CT, increased access to combination antiretroviral therapy and PCP prophylaxis may also alter the radiographic appearance of PCP [3]. Even in patients with inconsistent treatment adherence, the degree of immunosuppression, extent of opportunistic infection, and radiographic presentation of PCP may not be directly comparable to those with HIV who are treatment-naive.

In the case of the patient we describe, his two days of therapy with clindamycin and primaquine at the referring hospital could theoretically have altered his chest CT finding and explained the lack of ground glass opacities. However, this seems highly unlikely because (1) his plain films on admission to each institution were similar and (2) he worsened clinically after receiving the initial PCP-directed therapy. This worsening, which is often seen with PCP, suggests a robust alveolar and interstitial inflammatory response, which we would expect to cause more, not fewer, ground glass opacities on chest CT.

In summary, we describe a case of PCP in a patient with AIDS, with classic presenting symptoms but an unusual radiographic pattern on chest CT featuring nodular opacities and no ground glass. The lack of ground glass opacities resulted in brief PCP treatment interruption. CT scan findings alone should not be used to discontinue PCP-specific treatment, particularly in patients with a clinical syndrome that is otherwise consistent with PCP.

## Figures and Tables

**Table 1:** Initial laboratory studies.

Lab name	Value at outside hospital (4 days prior to admission)	Value on admission to our hospital (day 0)	Reference range
WBC (k/uL)	7.0	6.2	3.9–11.7
Neutrophils (%)	—	63	44–68
Lymphocytes (%)	—	19	25–44
Monocytes (%)	—	13	0–7
Eosinophils (%)	—	3	0–4
Bands (%)	12	0	0
Hemoglobin (g/dL)	10.3	10.1	13.3–17.7
Glucose (mg/dL)	293	71	70–199
Creatinine (mg/dL)	2.4	1.7	0.70–1.30
Troponin (ng/mL)	—	<0.04	<0.04
Lactate (mmol/L)	4.7	1.7	0.5–2.2
Lactate dehydrogenase (u/L)	—	385	100–190
Beta-D-glucan (pg/mL)	—	398	<60 negative
			61–79 indeterminate
			>80 positive
Cryptococcal antigen	—	Negative	Negative
Coccidioides antibody (complement fixation)	—	<1 : 2, Negative	Negative
Coccidioides antibody (immunodiffusion)	—	Negative	Negative
CD4+ T-cells (cells/uL)	—	15	420–1250
HIV viral load (copies/mL)	—	178,887	0

**Table 2:** Microbiology studies.

Test name	Hospital day collected	Result
Blood culture x2	0	Negative
Nasopharynx influenza RNA	0	Negative
Blood for acid-fast bacilli	1	Negative at 42 days
Induced sputum for acid-fast bacilli	2	Negative at 56 days
Induced sputum for acid-fast bacilli	3	Negative at 56 days
Induced sputum for MTb PCR	3	Negative
Induced sputum for bacterial culture	2	Few oronasal flora
Induced sputum for fungal culture	2	*Candida albicans* ^∗∗^
BAL fluid for PCP examination	4	Many clumps of trophozoites and cysts. No viral inclusions seen. No fungi seen.
BAL fluid for fungal culture	4	*Candida albicans* ^∗∗^
BAL fluid for acid-fast bacilli	4	Negative at 56 days
BAL fluid for bacterial culture	4	No organisms seen
Serum cryptococcal antigen	1	Negative
Coccidioides antibody (complement fixation)	1	<1 : 2, Negative
Coccidioides antibody (immunodiffusion)	1	Negative
Transbronchial biopsy for acid-fast bacilli culture	4	Negative at 28 days
Transbronchial biopsy for bacterial culture	4	Negative at 56 days

^∗∗^Patient had thrush, and this was felt to be an upper airway contaminant.
